# A reversed gender pattern? A meta-analysis of gender differences in the prevalence of non-suicidal self-injurious behaviour among Chinese adolescents

**DOI:** 10.1186/s12889-017-4614-z

**Published:** 2017-07-28

**Authors:** Xueyan Yang, Marcus W. Feldman

**Affiliations:** 1 Institute for Population and Development Studies, School of Public Policy and Administration, Xi’an Jiaotong University, #28 Xianning Xi Road, Xi’an, Shaanxi Province 710049 People’s Republic of China; 2 Morrison Institute for Population and Resource Studies, Stanford University, Stanford, CA USA; 3 Postal Address: Gilbert 116, Stanford University, Stanford, CA 94305 USA

**Keywords:** Reversed gender pattern, NSSI prevalence, Chinese adolescents, College students, Middle school students, Clinical samples

## Abstract

**Background:**

A reversed gender pattern has been observed in the suicide rate in China compared to elsewhere. Like suicidal behaviour, non-suicidal self-injurious (NSSI) behaviour is a health-risk behaviour. We examined whether a reversed gender pattern existed in the prevalence of NSSI.

**Methods:**

Online literature databases were searched for English and Chinese articles on NSSI behaviours among the Chinese. A meta-analysis with a random-effects model and a subgroup analysis were used to estimate the odds ratios of gender differences in NSSI prevalence among Chinese adolescents including college students, middle school students, and clinical samples, as well as rural, urban, and Hong Kong middle school students.

**Results:**

There was a male bias in NSSI prevalence among college students (OR = 1.56, 95% CI = [1.30, 1.87], *p* < 0.001), and a female bias among middle school students (OR = 0.83, 95% CI = [0.73, 0.94], *p* < 0.01), but there was no gender difference among clinical samples (OR = 0.88, 95% CI = [0.41, 1.89], *p* > 0.1). The NSSI prevalence among middle school students had a female bias in the rural (OR = 0.58, 95% CI = [0.47, 0.72], *p* < 0.001) and Hong Kong areas (OR = 0.91, 95% CI = [0.86, 0.96], *p* < 0.001), with the gender difference in NSSI prevalence in the Hong Kong areas being greater than in rural areas. No gender difference in NSSI prevalence was found in urban areas (OR = 1.01, 95% CI = [0.84, 1.22], *p* > 0.1) among middle school students.

**Conclusions:**

Our analysis indicated the existence of specific gender and age patterns in NSSI prevalence among Chinese adolescents. The sample type, age, and the areas that have different gender norms and culture could partly explain this pattern.

**Electronic supplementary material:**

The online version of this article (doi:10.1186/s12889-017-4614-z) contains supplementary material, which is available to authorized users.

## Background

Since the 1990s, the suicide rate in China has decreased greatly but a unique reversed gender pattern remains. Based on data from 1995 to 1999 from the Chinese Ministry of Health, it was estimated that the suicide rate among women in China was 25.9, which was higher than the rate of 20.7 among men [1]. According to data from the World Health Organization (WHO), compared to worldwide rates, the suicide rate (suicides per 100,000 people per year) in China in 2011 was relatively low; however, there was still a reversed gender pattern compared to other countries [2]. Thus, China is one of very few countries (others are Pakistan and Bangladesh, based on 2011 data from the WHO) with a higher suicide rate among women than among men: the suicide rate in men is 7.1 compared to 8.7 in women [2]. This reversed gender pattern in suicide rate is especially apparent in the younger population (15–34 years). Other similar studies have also noted this reversed gender pattern [3, 4].

Like suicidal behaviour, non-suicidal self-injurious (NSSI) behaviour, defined as direct, deliberate destruction of one’s own body without an intent to die [5], is a health-risk behaviour; similarities between these two behaviours also appear to exist [6–8]. Because adolescents aged 15–19 years are the group most likely to be involved in NSSI behaviour, most existing research has focused on them [9]. In studies from Western countries, NSSI is usually viewed as primarily a female behaviour, but this has changed recently [10]. Before 2000, most studies showed that the NSSI prevalence among female adolescents was 1.5 to 3 times that in male adolescents [11]. However, more recent studies have contrasting conclusions: some still insist on a gender difference in the NSSI prevalence among adolescents [12–14], while others do not find this gender difference [6, 8, 15–17]. Nevertheless, a recent meta-analysis of gender difference in NSSI prevalence, which examined some associated factors, found a female bias in NSSI prevalence among adolescents worldwide, and a greater gender difference in clinical samples than in community samples [18]. However, it is difficult to apply the findings from the above-mentioned meta-analysis to China, given the reversed gender pattern in the suicide rate observed in China. Studies of NSSI in China began after 2002 mainly among adolescents (including college students and middle school students, and covering rural and urban areas of mainland China and the Hong Kong area), and there has been no consensus on whether there is a gender difference in NSSI prevalence among Chinese adolescents.

We conducted a literature review of NSSI prevalence among Chinese people and used a meta-analysis to address the following questions. Is there a gender difference in NSSI prevalence among adolescents in China? If so, is there a female bias in NSSI prevalence among Chinese adolescents, or is there a reversed gender pattern as with the suicide rate in China compared to other countries? Could such factors as age, sample type, gender norms, or culture explain the gender difference in NSSI prevalence in Chinese adolescents? Answering these questions might be of great significance for enriching studies of NSSI in China and might be very important for the design of gender-specific intervention strategies for NSSI among Chinese adolescents.

## Methods

### Study design and outcomes

The data used in this study were from a literature search conducted from 15 February to 4 March 2016. In order to ensure the consistency of the search, only the first author was responsible for the whole search. The literature search included studies in both Chinese and English. The periodic full-text database of the China National Knowledge Infrastructure and of the China Networked Digital Library of Thesis and Dissertations jointly produced by Tsinghua University and Tsinghua Tongfang Company in Beijing, China were searched for Chinese literature. The Google Scholar database produced by Google Inc. in Mountain View, CA, USA and Stanford University Libraries were jointly searched for English literature. Since few studies of NSSI exist in China, we used key words in the initial search but did not set parameters for years, target groups, or methods of studies. With ‘自残’, ‘自伤’, and ‘自我伤害’ as the key words, we found 428 articles from journals and 22 digital dissertations in Chinese. With ‘self-harm + China’, ‘self-injury + China’, ‘self-cutting + China’, and ‘self-mutilation + China’ as the key words, we found 22 English articles in journals.

As our focus was on only the gendered NSSI prevalence among the general Chinese population, the only inclusion criterion used was of quantitative studies providing data by gender of NSSI behaviour among the general Chinese population. According to this criterion, the first exclusion criterion used was of studies that were not of the general population; therefore, the literature titles that included ‘animal’, ‘psychiatric patients’, ‘infantile autism’, ‘autistic disorder’, and ‘drug addicts’ were excluded. In total, 58 appropriate Chinese articles and 21 appropriate English articles were downloaded.

The second exclusion criterion was of non-quantitative studies or studies that did not provide data by gender of NSSI behaviour among the Chinese general population. According to this criterion, when the 79 articles were analysed further regarding the area of study, target groups, methods, variables, and data used, 42 were excluded. These included two papers reporting qualitative or case studies, 11 literature reviews, three that were not about China, three about suicidal intent, 18 that did not provide data according to sex, and five that used the same data as other selected articles.

**Fig. 1:**
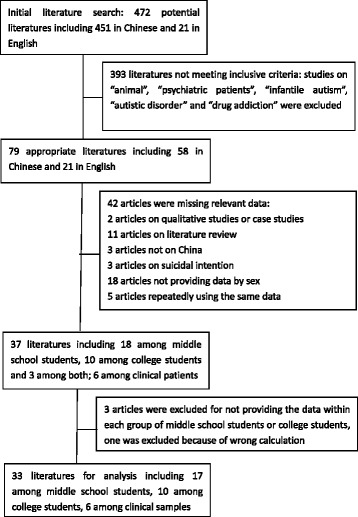
Flow chart of literature search and selection

Previous research shows that sample types matter for the gendered NSSI prevalence; [18] therefore, the third exclusion criterion used was of studies that did not clearly provide the data by sample types. According to this criterion, further analysis was conducted of the remaining 37 articles with respect to the specific target groups, which were found to be mainly the following: middle school students, college students, and clinical samples. Of these, ten focused on only college students and 18 focused on only middle school students (one provided data using an incorrect calculation; therefore, it was deleted), while three addressed both groups (these three were excluded because they did not differentiate between the two groups), and six focused on clinical patients (mainly adolescents and children). Thus, we were left with 17 articles that had middle school students as the target group, ten with college students as the target group, and six with clinical adolescents for our analysis. The flow chart of the literature search and selection process are in Fig. 1 and the included and excluded full-text literature is in Additional file 1: Appendix 1.

### Data extraction

Information about the authors and the titles, journals, investigation sites, time of the survey, measurements of NSSI behaviour, target groups, and cases of NSSI behaviour by gender was extracted from the selected papers. In order to ensure the quality of the extracted data, we conducted a parallel extraction; namely, two persons independently extracted data as required and a comparison was made between the two independent extraction results.

It was inferred from previous studies that age and the areas that have different gender norms or culture might be associated with the gender difference in NSSI prevalence [18, 19]. Therefore, besides the main information about the gendered NSSI prevalence, in the present study, where possible information about age of NSSI prevalence by gender and areas from where the samples came was extracted from the cited articles and previous research.

However, most of the literature cited in this study provided only a general age range of the samples, without providing the age information about the NSSI behaviours by gender. Therefore, only very limited information about the relationship between age and gendered NSSI prevalence was discussed and referenced.

In fact, economic and cultural systems differ between rural and urban areas in China. Generally, the level of economic development is relatively lower, and the culture and gender norms are generally more conservative and have more of a male preference, in rural areas than in urban areas [20]. This means that it might make sense to classify the samples into rural and urban groups. However, it is difficult to classify college students and clinical samples into rural or urban groups because all of the colleges and universities in China are located in urban areas, and all of the cited studies about college students and clinical adolescents did not provide enough data of the students’ or patients’ hometowns. Therefore, only the middle school students were classified into rural and urban groups according to the specific areas where the middle schools were located. Considering that there might be a big difference in social and cultural background between the urban areas in the mainland and Hong Kong, the areas of the studies were classified into three groups: rural areas, urban areas, and Hong Kong.

### Statistical analysis

Review Manager 5.3 [21] was used for the meta-analysis. The information about NSSI (e.g. cases, sample size) in all of the selected papers was input into the system using the subgroup analysis of the ‘intervention review’ function, and a random-effects model was used to estimate the odds ratio as the index of the effect size, with values less than 1 indicating females being more likely to report NSSI than males (i.e. odds of NSSI for men/odds of NSSI for women). The sex ratio in a population, such as the sex ratio at birth, is commonly calculated with the female population as the denominator; therefore, we used the odds of NSSI for women as the denominator for calculating the gendered odds ratio of NSSI; this is opposite to the calculation by Bresin and Schoenleber (2015) [18]. The confidence interval of each odds ratio was also provided.

The analysis strategy proceeded in two steps. First, it was tested whether the weighted odds ratio differed from 1 and whether there was significant heterogeneity in the effect size across studies of college students, middle school students, and clinical samples. Second, based on the very limited data extracted from all cited papers, we investigated whether the weighted odds ratio differed from 1 and whether there was significant heterogeneity in the effect size across studies in the rural, urban, and Hong Kong areas among middle school students.

## Results

Table 1 presents the results of the gender difference analysis of NSSI prevalence among college students, middle school students, and clinical samples. As shown in Table 1, there were ten studies covering 17,738 college individuals, of whom 9846 were boys and 7892 were girls. The odds ratio of the gendered NSSI prevalence among college students was 1.56, 95% CI = [1.30, 1.87], *p* < 0.001, indicating a male bias in NSSI prevalence among college students. There were 19 studies covering 80,448 middle school individuals, of whom 37,806 were boys and 42,642 were girls. The odds ratio of the gendered NSSI prevalence among middle school students was 0.83, 95% CI = [0.73, 0.94], *p* < 0.01, indicating a female bias in NSSI prevalence among middle school students. There were six studies covering 952 clinical patients, of whom 392 were male and 560 were female. The odds ratio of the gendered NSSI prevalence among clinical samples was 0.88, 95% CI = [0.41, 1.89], *p* > 0.1, indicating no gender difference in NSSI prevalence among clinical samples. In the total sample, there were 35 studies in total covering 99,138 individuals, of whom 46,090 were boys and 53,048 were girls. The odds ratio of the gendered NSSI in the total sample was 0.96, 95% CI = [0.84, 1.10], *p* = 0.56. The test for subgroup differences showed that the gender difference in NSSI prevalence varied significantly across the college, middle school, and clinical groups (χ^2^ = 31.05, *p* < 0.00001, I^2^ = 93.6%), and indicated that the classification of the three groups was reasonable. The significance of the heterogeneity in the three subgroups means that there might be other factors that need to be included to explain the gender difference in NSSI prevalence in these three subgroups.

**Table 1:** The gender difference in NSSI prevalence by sample types

	Number of studies	Men		Women		Odds Ratio	95% CI	Test of heterogeneity
		Events	Total	Events	Total			
College students	10	2425	7892	2304	9846	1.56	1.30,1.87***	Q(9) = 34.33, *p* < 0.0001
Middle school students^a^	19	8404	37,806	10,000	42,642	0.83	0.73,0.94**	Q(18) = 211.71, *p* < 0.00001
Clinical sample	6	392	392	560	560	0.88	0.41,1.89	Q(5) = 70.48, *p* < 0.00001
Total	35	11,221	46,090	12,864	53,048	0.96	0.84,1.10	Q(34) = 575.84, *p* < 0.00001
Heterogeneity: *τ* ^2^ = 0.14, *χ* ^2^ = 575.84, df = 34 (*p* < 0.00001), I^2^ = 94% Test of overall effect: Z = 0.59 (*p* = 0.56),								
Test for subgroup difference: *χ* ^2^ = 31.05, df = 2 (*p* < 0.00001), I^2^ = 93.6%								

*p < 0.05 for total effect, **p < 0.01 for total effect, ***p < 0.001 for total effect

^a^ There are 17 studies among middle school students, but one of which provided three groups of data (Yang, Han & Shao, 2013), therefore, there are 19 groups of data were included into the analysis

Table 2 presents the results of gender difference analysis in NSSI prevalence among middle school students in the rural, urban, and Hong Kong areas. As shown in Table 2, there were five studies covering 27,211 rural individuals, of whom 13,934 were boys and 13,277 were girls. The odds ratio of gendered NSSI prevalence among middle school students in rural areas was 0.91, 95% CI = [0.86, 0.96], *p* < 0.001, indicating a female bias in NSSI prevalence among middle school students in rural areas. There were eight studies covering 28,658 urban individuals, of whom 13,990 were boys and 14,668 were girls. The odds ratio of gendered NSSI prevalence among middle school students in urban areas was 1.01, 95% CI = [0.84, 1.22], *p* > 0.1, indicating no gender difference in NSSI prevalence among middle school students in urban areas. There were five studies covering 20,622 Hong Kong individuals, of whom 8041 were boys and 12,581 were girls. The odds ratio of gendered NSSI prevalence among middle school students in Hong Kong was 0.58, 95% CI = [0.47, 0.72], *p* < 0.001, indicating a female bias in NSSI prevalence among middle school students in Hong Kong. The gender difference in NSSI prevalence in the Hong Kong group was greater than in the rural group.

**Table 2:** The gender difference in NSSI prevalence by areas

	Number of studies	Men		Women		Odds Ratio	95% CI	Test of heterogeneity
		Events	Total	Events	Total			
Rural^a^	5	3247	13,934	3339	13,277	0.91	0.86,0.96***	Q(4) = 1.01, *p* = 0.91
Urban^a^	8	3669	13,990	3605	14,668	1.01	0.84,1.22	Q(7) = 56.32, *p* < 0.00001
Hongkong^a^	5	1066	8041	2386	12,581	0.58	0.47,0.72***	Q(4) = 21.49, *p* < 0.0003
Total	18	7982	35,965	9330	40,526	0.84	0.74,0.96*	Q(17) = 189.86, *p* < 0.00001
Heterogeneity: *τ* ^2^ = 0.06, *χ* ^2^ = 189.86, df = 17 (*p* < 0.00001), I^2^ = 91% Test of overall effect: Z = 2.58 (*p* = 0.010)								
Test for subgroup difference: 0.06, *χ* ^2^ = 17.35, df = 2 (*p* = 0.0002), I^2^ = 88.5%								

*p < 0.05 for total effect, **p < 0.01 for total effect, ***p < 0.001 for total effect

^a^ There are one from 19 groups of data among middle school students was excluded because this data included middle schools in both rural and urban areas, but did not provide the separated data

The test for subgroup differences showed that the gender difference in NSSI prevalence varied significantly among the rural, urban, and Hong Kong groups (*χ* ^2^ = 17.35, *p* = 0.0002, I^2^ = 88.5%), meaning that the classification of the groups was reasonable. However, the significance of the heterogeneity in the Hong Kong and urban groups indicates that there might be other additional factors that need to be included to explain the gender difference in NSSI prevalence in these two groups.

## Discussion

Studies of gender differences in health have shown that China’s gender pattern is different from that of other countries. For example, suicide rates in most countries are male-biased, with significantly higher rates in males than in females. China is the only country in which the suicide rate among females is higher than among males [1, 2]. The present study addressed whether China also shows this unique reversed gender pattern in NSSI prevalence among adolescents in community and clinical samples.

Our analysis indicated the existence of a unique gender pattern in NSSI behaviour among adolescents, with the NSSI prevalence among boys being higher than among girls, but only for college students. For middle school students, the NSSI prevalence followed the usual female bias, at least to some extent, which is in accordance with findings obtained from studies in Western countries [12–14]. Existing research in Western countries indicates that the gender difference in NSSI prevalence is greater among clinical samples than among community samples [18], while our analysis showed different results: there was no gender difference in NSSI prevalence among clinical samples. However, considering that the number of included studies of clinical samples was very limited (only six studies), this result should be interpreted with caution.

Most articles cited in the present study did not analyse gender differences in NSSI prevalence among Chinese adolescents. Only one study pointed out that there is a ‘boy crisis’ in the prevalence of NSSI behaviour among college students in China, and that gender role conflict could be an important cause of this phenomenon [22]. From previous studies, it remains difficult to infer possible causes of gender differences in NSSI prevalence among Chinese adolescents.

In studies from Western countries, biological and socio-environmental factors are commonly used to explain gender differences in NSSI behaviour. It has been suggested that gender differences in hormones (e.g. testosterone vs. oestradiol) might produce gender differences in NSSI behaviour [23]. On the other hand, gender role socialisation might make the female population more susceptible to NSSI behaviour [24, 25]. However, these studies in Western countries do not explain the unusual gender pattern of gender differences in NSSI prevalence among Chinese adolescents. We might still infer that biological factors and the different socio-environmental forces that adolescents of different genders face could contribute to gender differences. Thus, we could assume that the difference between boys and girls in physical development is the main reason for the female bias in these behaviours among middle school students and for the male bias in NSSI behaviour among college students, as girls enter puberty earlier than do boys [26, 27]. If this assumption is right, the gender difference in NSSI prevalence might vary across different age groups. From the socio-environmental perspective, China is a modified patriarchal society [28], which might underlie a gender role conflict in female middle school students and male college students [19], and lead to a higher prevalence of NSSI behaviour in these groups. If this assumption is correct, the gender difference in NSSI prevalence might vary across areas that have different gender norms and culture.

Unfortunately, most articles cited in this present study did not provide gendered data of NSSI prevalence by age, and only one study of children with a clinical diagnosis of self-inflicted ulcers provided gendered data by age, showing that the prevalence of self-inflicted ulcers was almost evenly distributed in boys and girls aged 2–5 years and 13–16 years, but was significantly higher among boys than girls aged 6–12 years [29]. This finding indirectly suggests that age might have some relationship with gendered NSSI prevalence among Chinese adolescents, but more data is needed to determine what this relationship is and how it works, and more biological factors should be considered in future studies.

Our results concerning the relationship between the areas and gendered NSSI prevalence suggested a female bias in NSSI prevalence in rural areas, whereas no gender difference was found in NSSI prevalence in urban areas, which partly supports the assumption of the influence of culture and gender norms. The patrilineal culture is stronger in rural areas than in urban areas and girls might face more pressure from gender role conflicts, which might induce a female bias in NSSI in rural areas [30]. While the results for the Hong Kong area tell another story, there is still a female bias in NSSI prevalence in Hong Kong and this gender difference is even greater than in the rural areas of the mainland. A possible explanation is that the socio-economic development and cultural background of Hong Kong is more similar to that of Western countries; therefore, the gender pattern in NSSI prevalence might be more similar to that of Western countries than to that of the urban areas of the mainland [31]. The significant heterogeneity in the urban areas of mainland China and Hong Kong indicates that there might be other factors that should be included to explain the gender patterns in NSSI prevalence in these two areas.

## Conclusion

In summary, this is the first meta-analysis to address the reversed gender pattern of NSSI prevalence among Chinese adolescents. Two conclusions can be drawn from our analysis. First, the NSSI prevalence among Chinese adolescents showed specific gender and age patterns, with a male bias in NSSI prevalence among college students, a female bias in NSSI prevalence among middle school students, and no gender difference in NSSI prevalence among clinical samples. Second, the very limited data from previous research on the relationship between age and gendered NSSI prevalence partly supported the assumption that biological factors might explain the gender difference in NSSI prevalence among Chinese adolescents, while the subgroup analysis of gendered NSSI prevalence across the rural, urban, and Hong Kong areas partly supported the assumption that socio-environmental factors might explain the gender difference in NSSI prevalence among Chinese adolescents. These findings can significantly enrich existing studies of NSSI across various societies and cultures. Furthermore, the findings emphasise the need to develop a gender-specific intervention strategy for NSSI among Chinese adolescents at different stages of development.

Although the age of the sample was not restricted in our search for articles on NSSI behaviour, the target groups of most studies were mainly college students or middle school students. Further study is warranted as to whether the different gender patterns we found among adolescents of different ages are maintained among adults of different ages. While the studies we analysed involved gender differences in NSSI behaviour, we addressed only very limited factors associated with these gender differences among Chinese adolescents, because most articles in our meta-analysis neglected this gender difference and could not provide reasonable interpretations of it. Future studies could survey larger numbers of Chinese people of different genders and ages to explore the interactions between gender and age in NSSI behaviour.

## Additional file

Additional file 1: Appendix 1. The included and excluded literature. (DOCX 22 kb)
