# Supplementary material for: A reversed gender pattern? A meta-analysis of gender differences in the prevalence of non-suicidal self-injurious behaviour among Chinese adolescents
Source: BMC Public Health. 2017 Jul 28;18:66. doi: 10.1186/s12889-017-4614-z (PMC5534103; doi:10.1186/s12889-017-4614-z)
Supplement: Additional file 1: Appendix 1. — The included and excluded literature. (DOCX 22 kb) [file 12889_2017_4614_MOESM1_ESM.docx]

**Additional file 1**

**Appendix 1: The included and excluded literature**

**Inclusion Criterion:** Quantitative studies providing data by sex of NSSI behaviour among the general Chinese population.

**Included Studies**

**Target group: College students**

[1] Qiao, HF.& Chen, Y. (2012). An investigation of non-suicidal self-injury and coping styles in undergraduates, *Journal of Psychiatry (in Chinese)*, 6(6): 436-439

[2] Liu, ZJ & Chen, P. (2012). A comparison study on self-harm behaviors between students in sport majors and non-sport majors in Beijing Normal University, *Sports (in Chinese)*, 6: 77-79 [3] [3]You. JN., Zhong, J. & Liang, YJ. (2013). Non-suicidal self-injury and its influencing factors among college students in Beijing, *China Journal of School Health (in Chinese)*, 34(5): 559-564

[4] Li, YY. & Meng, XH. (2014). Ｌearning pressure and self-injurious behavior in vocational schoolstudents: a correlation study, *Qilu Medical Journal (in Chinese),* 6: 540-542

[5] Pan, H., Huang, RZ. & Tu QM. (2014). Correlation between self-injury and coping style of Hunan’s tuition-free normal college students, *Sichuan Mental Health (in Chinese)*, 27(1): 22-24

[6] Wang, SJ. (2010). A survey on associated factors with self-harm among college students, *a master degree thesis of Taishan Medical School(in Chinese*), 2010

[7] Wang, PX., Lu, YY. & Li, P. (2007). A study on the epidemiological investigation of deliberate self-harm among college students, *Henan Medical Research (in Chinese),* 3(3): 256-257.

[8] Wang, TT. (2013). Self-injurious behaviors and psychological determinants in college students, *a master degree thesis of Anhui Medical University (in Chinese)*

[9] Tao, SM. Wu, XY., Liu, Y.H. Zhang, MK., Zhang, S.C. & Tao, FB. (2014). Self-harm in medical college students and its relation with mobile phone dependence and depressive symptoms, *China Journal of Psychological Health (in Chinese),* 6(6): 472-477

[10] Chao, Q., Yang, X., & Luo, C. (2015). Boy crisis? sex differences in self-injurious behaviors and the effects of gender role conflicts among college students in china.*American Journal of Men’s Health.*

**Target group: Middle school students**

[1] Tang, J., Ma, Y., Guo, Y., Ahmed, N. I., Yu, Y., & Wang, J. (2013). Association of aggression and non-suicidal self injury: a school-based sample of adolescents.*Plos One,* 8(10), e78149.

[2] Xu, ZW. (2011). The self-injurious behaviors and its influencing factors among secondary school students in the countryside of Anhui Province, *a master degree thesis of Anhui Medical University (in Chinese)*

[3] Sun, Y. Tao, FB., Xu, SJ., Zhu, P., Huang, K. & Su, PY. (2008). Self-injurious behaviors and psychosocial factors among rural middle school students in Anhui Province, *China Journal of School Health (in Chinese)*, 5(5): 424-427.

[4] Yang, DM., Han, WM. Shao, CX & Su, XX. (2013). Surveillance for self-injury and suicide among middle school students in Beijing Tongzhou District during 2008-2012, *China Journal of School Health (in Chinese),* 12(12): 1102-1105.

[5] Xuan, PX. (2014). Study on the relationship between emotion dysregulation and self-harm behaviors of junior school students (in Chinese), *a master degree thesis of Henan University*

[6] Wang, L. (2011). The epidemiological survey and correlation analysis about deliberate self-harm behavior of junior school students in Dalian *(in Chinese)*, *a master degree thesis of Dalian Medical University*

[7] Lei, XL., Xu, SJ., Liu, XY., Zu, P., Zhang, SC. & Zhao, YQ. et al. (2012). Relationship of self-harm and suicidal behavior among middle school students, *China Journal of School Health (in Chinese)*, 33(4): 393-395.

[8] Kong, XJ., Liu, Y., Zhao QH. Yuan, L & Luo, L. (2014). A study on the correlation between alienation and self-harm among left-behind junior middle school students in rural areas, *China Western Science and Technology (in Chinese)*, 2: 124-125

[9] Zhang, JX. (2011). A study on self-harm behaviors and associated factors, *a master degree thesis of Shandong University (in Chinese).*

[10] Xiao, YN. (2009). Self-injurious behaviors in adolescents with repeated childhood abuses experiences, *a master degree thesis of Anhui Medical University (in Chinese).*

[11] Fu, JL., Wan, YH. Sun, Y. Tao, SM., Zu, P & An, J et al., (2013). Relation of screen time and psychological sub-health to self-harm behavior in adolescents, *Chinese Mental Health Journal (in Chinese)*, 37(6): 468-472.

[12]You, J., Leung, F., Lai, C. M., & Fu, K. (2012). The associations between non-suicidal self-injury and borderline personality disorder features among Chinese adolescents. *Journal of personality disorders,* 26(2), 226-237.

[13]Law, B. M. F., & Shek, D. T. L. (2013). Self-harm and suicide attempts among young Chinese adolescents in Hong Kong: prevalence, correlates, and changes, *Journal of pediatric and adolescent gynecology,* 26(3), S26-S32.

[14]You, J., Leung, F., & Fu, K. (2012). Exploring the reciprocal relations between nonsuicidal self-injury, negative emotions and relationship problems in Chinese adolescents: a longitudinal cross-lag study. *Journal of abnormal child psychology,* 40(5), 829-836.

[15]Wong, J. P., Stewart, S. M., Ho, S. Y., & Lam, T. H. (2007). Risk factors associated with suicide attempts and other self‐injury among Hong Kong adolescents. *Suicide and Life-Threatening Behavior,* 37(4), 453-466.

[16]Cheung, Y. T. D., Wong, P. W. C., Lee, A. M., Lam, T. H., Fan, Y. S. S., & Yip, P. S. F. (2013). Non-suicidal self-injury and suicidal behavior: prevalence, co-occurrence, and correlates of suicide among adolescents in Hong Kong. *Social psychiatry and psychiatric epidemiology,* 48(7), 1133-1144.

[17] Yaun, BC. Wang, H., Li, JQ. & Wu, R. (2015). Correlation between adolescent sub-health and self-injury behavior among middle school students in three gorges area of Chongqing, *Modern Preventive Medicine (in Chinese),* 6: 1042-1044

**Target group: Clinical patients**

[1] Sun GR & Wang, YX (2011). An analysis on motivation for self-injurious behaviors among 131 cases among college students, *China School Medicine (in Chinese),* 25(11), 812-813.

[2]Wang J.H. & Song, LL .(2000). 204 case reports on non-suicidal self-injury, *Chinese Journal of Behavioral Medicine and Brian Science (in Chinese),* 9(2), 155-155.

[3]Xu, XM (2013).A study on genetic polymorphism of Self-injurious behavior among youth people (in Chinese) , *A Doctoral Dissertation submitted to Chongqing Medical University*

[4]Tang, JH, Wang, GH, Wang, XP, Bai XG, Weng, SH & Liu ZC (2005).an analysis on factors associated with self-injurious behaviors among depression adolescents, *Chinese Mental Health Journal (in Chinese),* 19(8), 536-538.

[5] Cheng, XT. (1987). Pay attention on psychological health education among youth people: an analysis on self-injurious patients who took poison, *Chinese Mental Health Journal (in Chinese),*2: 84-64.

[6] Wang, X & Yin, C. (2011). A clinical analysis on factors associated with self-injurious anabrosis and psychological behaviors among children, *Modern Hospital (in Chinese),* 11(1), 9-10.

**Excluded Literature**

**Exclusion reason:** **Qualitative study or case study**

[1] Tong, KM (2007). An analysis and intervention on self-injury among higher vocational students, *Health Medicine Research and Practice in Higher Institutions,*4(3): 52-54.

[2] Hu, HY (2014). A study on self-schema of self-injury among college students (in Chinese), *master degree thesis submitted to Central China Normal University*

**Exclusion reason: Literature review**

[1]Muehlenkamp, J. J., Claes, L., Havertape, L., & Plener, P. L. (2012). International prevalence of adolescent non-suicidal self-injury and deliberate self-harm. *Child & Adolescent Psychiatry & Mental Health,* 6(1), 10.

[2]Phillips, M. R. (2005). Understanding the unique characteristics of suicide in china: national psychological autopsy study. *Biomedical & Environmental Sciences,* 18(6), 379-389.

[3]Portzky, G., & Van, H. K. (2007). Deliberate self-harm in adolescents. *Current Opinion in Psychiatry,* 20(4), 337-342.

[4]Chan, J., Draper, B., & Banerjee, S. (2007). Deliberate self-harm in older adults: a review of the literature from 1995 to 2004.*International Journal of Geriatric Psychiatry,* 22(8), 720-32.

[5]Xu Y, Chen, J & Deng, LP (2010). The intervention of family and social work on self-harm behaviors, *The Monthly Journal of Social Work (in Chinese),* (18): 24-26

[6] Huang, RZ, Ding, LP & Huang, M. (2013). The status, associated factors and intervention on NSSI behaviors, *Chinese Journal of Clinical Psychology (in Chinese),* 21(6), 965-967.

[7] Zhang, AH & Tao, FB. (2008). Self-harm behaviors among adolescents, *Modern Preventive Medicine (in Chinese),* 35(11), 2032-2034.

[8]Wang, ZL, Zhang, WJ & Zheng, AM (2008). A multi-factor analysis on self-mutilation among adolescents, *Chinese primary health care (in Chinese)*, 22(12), 68-70.

[9] Chen, J & Su, PY (2015). Brief introduction on Lewis Mahdy & Michal et al.’s study: the state of health information obtained through online searches for self-injury, *Chinese Journal of School Health (in Chinese),* 2: 164

[10] Chen, J & Su, PY (2015). Brief introduction on Mars, Heron & Crane’s study: the clinical and social outcome of adolescent self-harm: Population based birth cohort study, *Chinese Journal of School Health (in Chinese),* 2: 181.

[11] Chen, J & Su, PY (2014). Brief introduction on Geulayov, Metcalfe & Heron’s study: parental suicidal attempt and off-spring self-harm and suicidal thoughts: results from Avon longitudinal Study parents and children (ALSPAC) birth cohort, *Chinese Journal of School Health (in Chinese),* 8:1

**Exclusion reason: Did not provide data by sex**

[1]Chu, SJ, Dong, SP, Yang, M. Huang, ZC & Lin XH .(2012). An analysis on the coping style of family factors and correlation with suicidal intention among college students, *Chinese Journal of School Health (in Chinese),* 33(10).1254-1255

[2]Kong, XJ, Liu Y & Liu WH. (2014). Analysis on correlation between social support and self-harm, *Nursing Research (in Chinese),* 26:3240-3241.

[3]Liang HH, Xu X, Liu, M, Wang, YX & Ma, CX (2012). A survey on impacts of family education on self-mutilation, *Academic Journal of Shaanxi Youth Professional College (in Chinese),* (3): 53-56.

[4]Tan, Y .(2009).Causes and Intervention on self-injurious behaviors among middle school students in Liupanshui city, *Academic Journal of Liupandhui Normal College,* 21(2):76-78.

[5]Xu, Y & Ma, L.(2013). A study on characteristics and causes of self-harm behaviors among rural left-behind adolescents: based on a survey in Macheng City of Hubei Province, *Academic Journal of South-Central University of Nationalities (in Chinese)*, 33(4), 90-96.

[6]Xu, ZW, Su, H, Wu, JL, Chang, WW & Sun YH (2010). The relationship between self-harm behaviors and locus of control among rural left-behind middle school students, *Chinese Journal of Public Health (in Chinese),* 26(7): 868-869.

[7]Wang, YH, Zhang, W, Peng, JX, Mo, BR & Xiong, S (2009). The relationship between parent-child attachment, self-concept and self-harm among college students, *Psychological Exploration (in Chinese),*29(5), 56-61.

[8]Feng, Y.(2008). The relationship between the self-harm behaviors and individual emotional factors and family environment factors (in Chinese), *Doctoral Dissertation submitted to Central-China Normal University.*

[9]Ma, MQ, Tan, H, Wang, W, Wang, C & Jin R (2013). The impacts of ability for solving social problems on self-harm status among left-behind women in nationality region, *Theory Learning (in Chinese)*, (30), 95-96.

[10] Yang, LH, Ma XL & Zhang, H .(2015). The impacts of neglected childhood on self-harm behaviors among junior high school students: the intermediate effects of social support, *Educational Theory and Practices (in Chinese, )*( 2):17-19

[11]Zhang, F. Cheng, WH, Xiao, ZP & Liu, WM (2015). A study on reliability and validity of Chinese version of Ottawa self-injury inventory, *Academic Journal of Shanghai Jiaotong University (Medical edition) (in Chinese),*3:460-464

[12] Zheng, Y. (2006). An epidemiological survey and functional model among middle-school students in Wuhan City (in Chinese), *Doctoral Dissertation submitted to Central-China Normal University*

[13]Wang, L & Wang, D. (2009). A study on self-harm behaviors among middle school students and its associated factors, *Chinese Journal of Health Psychology (in Chinese).*17(3), 314-316.

[14]Xu, Y. (2014). The self-injurious adolescents’ situational cognition (in Chinese), *A Doctoral Dissertation submitted to Central-China Normal University.*

[15]Leung, S. W., & Leung, F. (2009). Construct validity and prevalence rate of borderline personality disorder among Chinese adolescents. *Journal of Personality Disorders,*23(5), 494-513.

[16]You, J., Lin, M. P., & Leung, F. (2013). Functions of non-suicidal self-injury among Chinese community adolescents. *Journal of Adolescence,*36(4), 737-45.

[17] Xu, YQ, Fei, LP, Chen, QH, Wang, LW, Li CB & Sun XH (2012). Correlation between clinic situation on self-injurious patients and weather in one hospital of Shanghai during 2007-2010, *Journal of Psychological Medicine (in Chinese),* 25(1), 15-17.

[18]Li XY, Xu, LR, Ding, K, Zhang, XJ & Wang, HF (2008). A study on self-injurious and suicidal cases on clinic reception in an emergency ward, *Strat Journal of Preventive Medicine (in Chinese),* 14(3), 36-38.

**Exclusion reason: Suicidal intent**

[1]Chen, H., Mishara, B. L., & Liu, X. X. (2010). A pilot study of mobile telephone message interventions with suicide attempters in china*. Crisis,*31(2), 109-12.

[2]Tsoh, J., Chiu, H. F., Duberstein, P. R., Chan, S. S., Chi, I., & Yip, P. S., et al. (2005). Attempted suicide in elderly Chinese persons: a multi-group, controlled study. *American Journal of Geriatric Psychiatry,* 13(7), 562-71.

[3]Chiu, H. F. K., Lam, L. C. W., Pang, A. H. T., Leung, C. M., & Wong, C. K. (1996). Attempted suicide by Chinese elderly in hong kong. *General Hospital Psychiatry,*18(6), 444-7.

**Exclusion reason: Not about China or Chinese people**

[1]Casillas, A., & Clark, L. A. (2002). Dependency, impulsivity, and self-harm: traits hypothesized to underlie the association between cluster b personality and substance use disorders.*Journal of Personality Disorders,*16(5), 424-36.

[2]Hempstead, K. (2006). The geography of self-injury: spatial patterns in attempted and completed suicide. *Social Science & Medicine,*62(12), 3186-96.

[3]Hawton, K., & Harriss, L. (2008). The changing gender ratio in occurrence of deliberate self-harm across the lifecycle. *Crisis,*29(1), 4-10.

**Exclusion reason: Did not provide data by sample types**

[1]Tang, J., Yu, Y., Wu, Y., Du, Y., Ma, Y., Zhu, H., ... & Liu, Z. (2011). Association between non-suicidal self-injuries and suicide attempts in Chinese adolescents and college students: a cross-section study.*PloS one,* 6(4), e17977.

[2] Zhang, AH, Tao, FB & Su PY (2008). An analysis on self-harm behaviors and its associated factors, *China Public Health (in Chinese),* 24(11):1303-1305.

[3] Fang, YR, Fu, LJ, Wang, JL, Guo, TY, Xing, C, Huang, ZH & Tu CY.(2011). The correlation between the self-mutilation behaviors and sub-health status among college and middle-school students in Shaoxing City, *Chinese Journal of School Health (in Chinese)*, 32(5), 579-581.

**Exclusion reason: Repeated data use**

[1] Wang, L, Sun, YJ, Lin, Y, Jin, X. Li, XH, Liu, Y & Liang, J. (2014). An analysis on the risk factors of self-harm behaviors among junior middle school students, *Academic Journal of Dalian Medical University (in Chinese),,* 36(5): 445-451

[2] Tang, J, Ma, Y, Guo, Y. Liu, HY, Xing, YF, Chen, YL & Yu YZ .(2014).the correlation between the emotional management and self-injurious behaviors among middle school students in Guangdong Province, *Chinese Journal of School Health (in Chinese),* 35(7): 967-969.

[3] Tang, J, Ma, Y, Guo, Y. Liu, HY, Xing, YF, Chen, YL & Yu YZ .(2014).An analysis on social and psychological factors associated with self-harm behaviors among middle school students, *Chinese Journal of School Health (in Chinese),* 35 (6):806-808

[4] Xiao YN, Tao, FB, Xu, SJ, Su, PY & Huang ZH (2008). The relationship between childhood abuse and self harm behaviors among adolescents, *China Public health (in Chinese),*24(9):1028-1030.

[5]You, J., & Leung, F. (2012). The role of depressive symptoms, family invalidation and behavioral impulsivity in the occurrence and repetition of non-suicidal self-injury in Chinese adolescents: A 2-year follow-up study. *Journal of adolescence,*35(2), 389-395.

**Exclusion reason: Wrong calculation**

[1] Xie, YB, Peng, ZW & Xu LP (2010). The correlation between self-injurious behaviors and internet addiction among middle school students, *Chinese Journal of Psychological Health (in Chinese),* 24(6): 469-472.
